# Optical, Electrical, and Structural Properties of NiO Thin Films, Derived by Sol–Gel Method

**DOI:** 10.3390/gels11120944

**Published:** 2025-11-24

**Authors:** Tatyana Ivanova, Antoaneta Harizanova, Nikolay Petkov

**Affiliations:** 1 Central Laboratory of Solar Energy and New Energy Sources, Bulgarian Academy of Sciences, Tzarigradsko Chaussee 72, 1784 Sofia, Bulgaria; 2 Physical Sciences, Munster Technological University, Rosa Avenue, Bishopstown, T12 P928 Cork, Ireland

**Keywords:** sol–gel, NiO, structure, optical properties

## Abstract

NiO films were successfully deposited by sol–gel spin coating on Si, glass, and ITO-covered glass substrates. The impact of the film thickness (the different number of layers), annealing temperatures (from 300 to 500 °C), and the substrate type on the crystal structure, film morphology, optical, and vibrational properties was investigated. X-ray diffraction (XRD) revealed a polycrystalline structure and the appearance of the cubic NiO phase. Field Emission Scanning Electron Microscopy (FESEM) was applied to explore the surface morphology of NiO films, deposited on glass and ITO substrates. The oxidation states of Ni were determined by X-ray photoelectron spectroscopy (XPS). The presence of Ni^2+^ and Ni^3+^ states was supposed. UV–VIS–NIR spectroscopy revealed that NiO films possessed a high average transparency of up to 74.6% in the visible spectral range when they were deposited on glass substrates, and up to 76.9% for NiO films on ITO substrates. The thermal treatments and the film thickness slightly affected the film transparency in the spectral range of 450–700 nm. The work function (WF) of the samples was determined. This research showed that good properties of sol–gel NiO films can be compared to the properties of those films produced using complicated and expensive techniques.

## 1. Introduction

Nickel oxide (NiO) gains outstanding scientific interest due to its remarkable chemical and thermal stability, as well as its excellent optical, electrical, magnetic, and electrochemical characteristics [1,2]. Stoichiometric nickel oxide is a Mott–Hubbard insulator and an antiferromagnetic material due to the fact that NiO has a resistivity in the order of 10^13^ Ω·cm [3]. To obtain stoichiometric NiO in thin film form is a very challenging task [4]. The electrical, optical, and magnetic properties of NiO films are strongly influenced by the deviation from ideal stoichiometry, which induces the formation of oxygen vacancies and/or nickel interstitials [4]. As one of the few p-type metal oxides, the p-type conductivity of NiO is due to nickel vacancies and/or interstitial oxygen. These defects act as shallow acceptors [5,6]. NiO is reported to show a low electron affinity with the reported values, ranging from 1.46 to 1.85 eV [7]. It is a wide bandgap semiconductor with a direct bandgap (the values are in the range of 3.6–4.0 eV), and it possesses good transparency (>60%) in the visible spectral region [6].

Considering the remarkable intrinsic properties of NiO, this material is widely used in photovoltaics, electrochromic-efficient devices, ultraviolet photodetectors, and sensors for detecting various gases [8]. There are three main applications of NiO films in solar cells: as a transparent conducting oxide (TCO), as a hole transporting layer (HTL) in perovskite photovoltaic devices, and as a p-type layer in the formation of a p-n heterojunction for transparent solar cells with the combination of an n-type layer. NiO is investigated as a prospective p-type TCO due to its NaCl-type structure, wide bandgap energy, crystallinity, and broad-spectrum range of transparency [9]. NiO films are proven to be successful as a hole transport layer in perovskite solar cells because of their chemical stability, high hole mobility, and relatively low fabrication cost [10]. The hole transport layer (HTL) plays an essential part in the performance of perovskite solar cells. The HTL efficiently collects and directs holes from the perovskite layer to the anode, and at the same time, it blocks electrons from reaching the anode [11]. The other important role is to protect the perovskite from external influences such as moisture, heat, and oxygen [11]. The fabrication of p-n heterojunctions with ZnO or TiO_2_ thin films can be applied in transparent photovoltaics [12] and UV photodetectors [13]. The transparent photovoltaic cell (TPV) is a solar cell that works by absorbing UV light (which is harmful) to generate electric power while passing the visible range light. The theoretical efficiency of heterojunction transparent solar cells was determined to be 21% [14]. Wide bandgap metal oxide semiconductors with high visible transmittance can be used to develop TPV that absorbs ultraviolet radiation by forming p-n heterojunctions [14].

Nickel oxide is one of the most studied anodic electrochromic materials. Nickel oxide is used in electrochromic devices because it can transform from a transparent state to a brownish-absorbing state when it is oxidized by applying a positive electrical potential, and NiO returns to the transparent state by applying a negative potential. NiO films possess a complementary effect on cathodic coloration films [15]. For example, when WO_3_ gains electrons and turns bluish (cathodic reduction), NiO becomes brown in color when it loses electrons (anodic oxidation) [15]. The excellent electrochromic properties of NiO include a high optical contrast, long memory effect, and a relevant voltage range, which makes nickel oxide films a suitable and excellent candidate for large-scale commercial application in smart windows and electrochromic displays [15].

The properties and the applications of NiO films are affected by the deposition techniques and the main precursors that are employed during the process. Different physical and chemical methods have been used to fabricate NiO films, such as pulsed-laser deposition (PLD), magnetron sputtering, thermal or electron beam evaporation, spray pyrolysis, chemical vapor deposition (CVD), etc [16,17,18]. Among these methods, the sol–gel approach is versatile, efficient, cost-effective (relatively low processing temperature, low-cost equipment), and allows control of the homogeneity, the microstructure, and the thickness of the obtained thin films [19]. Sol–gel coating deposition is widely used for preparing NiO thin films. Their optical and structural properties are highly dependent on the precursor‘s molarity [20,21], annealing temperature, deposition ambient [22], solvent type, and annealing ambient [20,23]. Nickel acetate is the most used precursor [20,21], but nickel nitrate [22] and nickel chloride [24] are also applied as Ni sources in sol–gel processing.

In our earlier report [25], we presented the deposition and the study of one-layered NiO films, deposited by the sol–gel spin-coating method on silicon and glass substrates. FTIR and XPS techniques were used for investigating NiO films obtained on Si wafers. NiO films on glass substrates were characterized by UV–VIS spectroscopy and a four-point probe, and their optical and electrical properties in dependence on the annealing temperatures (ranging from 200 to 500 °C) were reported [25].

In this work, NiO thin films were synthesized using sol–gel deposition. Their structural, morphological, optical, and electrical properties were investigated with respect to the substrate used (Si, glass, and ITO-covered glass), the number of layers, and thermal treatments. The obtained results in the present study have yielded new data, enhancing the understanding of the properties of NiO films.

## 2. Results and Discussion

### 2.1. XRD Analysis

**Figure 1:**
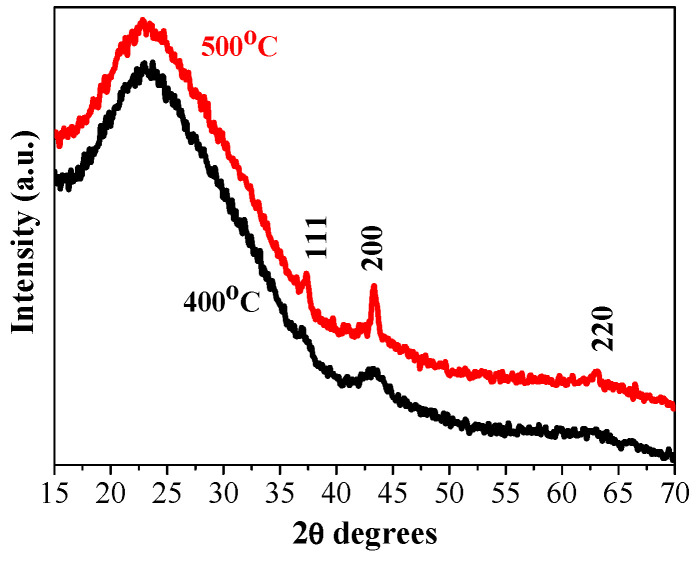
XRD patterns of the three-layered NiO films obtained on a glass substrate and annealed at 400 °C (black line) and 500 °C (red line).

Figure 1 shows XRD patterns of the NiO films with three layers, deposited on glass substrates and annealed at temperatures of 400 and 500 °C. The intense and very wide peak in the 2θ (two theta) range of 20–35° originates from the glass substrate. The XRD pattern of 400 °C-treated NiO film reveals a wide and very weak peak at 37.21° and a clear line at 43.13°, corresponding to the (111) and (200) planes, respectively.

This suggests that the crystallization is beginning along with the presence of an amorphous fraction. The observed XRD peaks are matched well with the standard XRD pattern (PDF card 00-047-1049) of the cubic NiO phase. This proves that the sol–gel film has a polycrystalline structure. Increasing the annealing temperature up to 500 °C, three stronger diffraction peaks corresponding to the (111), (200), and (220) orientations are manifested in the XRD pattern of the NiO film grown on a glass substrate (Figure 1, red line). The film crystallinity is improved by increasing the annealing temperature. Using the Scherrer formula [26], the crystallite sizes of the NiO films on glass substrates are determined, and the obtained values are presented in Table 1. The crystallite size becomes bigger with the increase in annealing temperatures. It is also observed that the crystallites grown along the (200) plane are greater than the crystallites along (111) for the case of the three-layered NiO films obtained on glass substrates.

**Figure 2:**
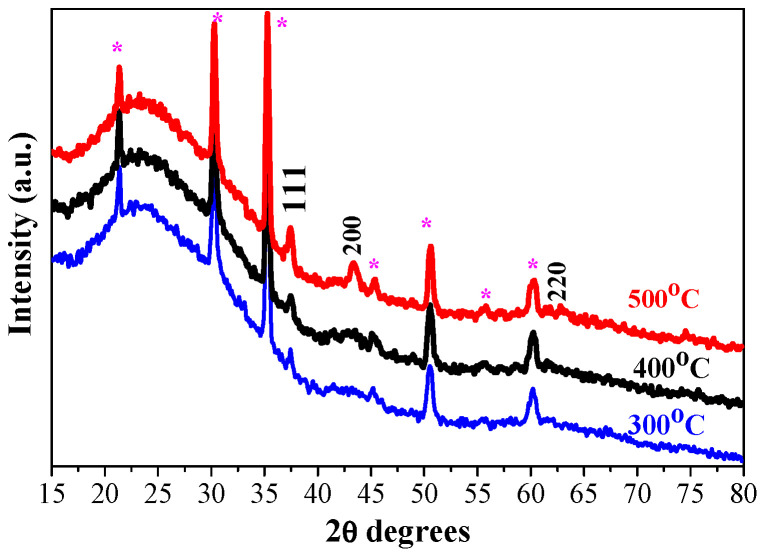
XRD patterns of sol–gel three-layered NiO films, obtained on ITO substrates. The films were annealed at 300 (blue color), 400 (black line), and 500 °C (red color). The asterisk symbol (*) indicates XRD lines, originating from the ITO substrate.

Figure 2 presents XRD patterns of the three-layered NiO films, deposited on ITO-covered glass substrate after the thermal treatments at 300, 400, and 500 °C. The characteristic XRD diffractions due to ITO-coated substrates are seen and marked as the lines that correspond to the reference peaks (PDF card 01-083-3550). NiO films, obtained on an ITO substrate, are crystallized in the cubic crystal phase. After 300 °C annealing, the XRD pattern exhibits a peak due to the (111) plane and a very broad and weak band around 45.2°. This finding suggests that the sol–gel NiO film, deposited on the ITO substrate, is preferentially crystallized in the (111) orientation. The broad line indicates the presence of a certain amorphous fraction. Actually, the (111) preferential orientation is found for the three-layered NiO films on ITO substrates (see Figure 2), annealed at the three different temperatures. The thermal treatments at 400 and 500 °C induce the presence of the two intense XRD peaks, assigned to the (111) and (200) planes, and a weak feature due to the (220) plane of the cubic NiO phase. The crystallization is enhanced. The crystallite sizes are estimated and given in Table 1.

**Figure 3:**
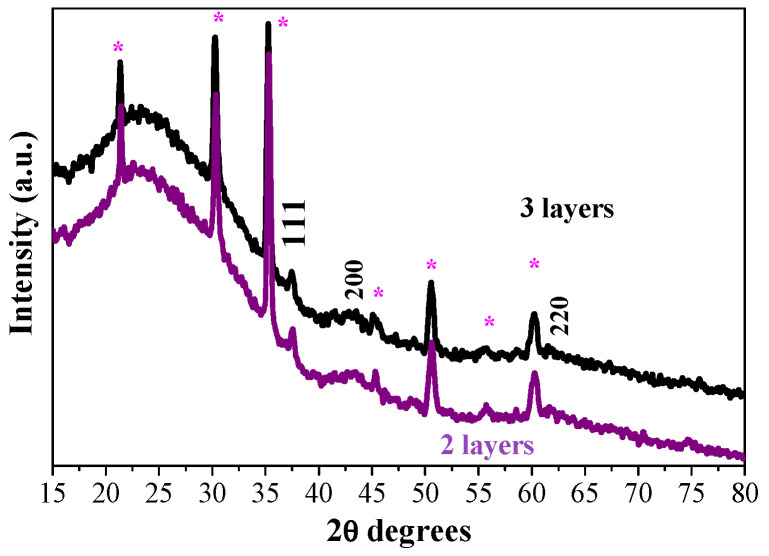
XRD patterns of the sol–gel NiO films with two (purple line) and three layers (black line), obtained on ITO substrates. The films are annealed at 400 °C. The asterisk symbol (*) indicates XRD lines, originating from the ITO substrate.

The effect of the number of layers on the film structure is studied. The XRD pattern of the two-layered sample is shown in Figure 3, and it is compared with the diffraction pattern of the three-layered NiO film. The films are annealed at 400 °C. The strongest line is observed at 2θ = 37.52° and is assigned to the (111) plane. This line is stronger and sharper in the diffraction pattern of the two-layered film. Accordingly, the estimated crystallite size is a little bigger (Table 1).

The substrate type influences the crystallization evolution of sol–gel NiO films. As can be deduced from Figure 1, nickel oxide film prefers to grow in the (200) orientation when it is deposited on a glass substrate. A rearrangement is observed for the sol–gel NiO films deposited on ITO substrates, as the XRD line, attributed to the (111) plane, is more pronounced and sharper. It is stated that the (200) orientation relates to the close-packing direction of the cubic structure, whereas the (111) plane corresponds to the pure compact plane of oxygen. The change of the film growth from the (200) to (111) orientation can be referred to the excess oxygen arrangement in the NiO lattice [27,28]. The different crystalline structures of the sol–gel NiO films, obtained on different substrates, will affect the optical properties.

### 2.2. XPS Characterization

XPS analysis provides information about the elemental composition, valence state, and oxidation state of a material. The study is focused on XPS analysis of the three-layered NiO films deposited on the ITO substrate, treated at the annealing temperatures of 300 and 500 °C.

**Figure 4:**
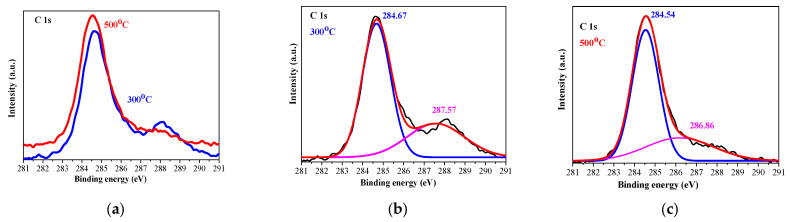
XPS spectra of the C 1s region of 300 (blue line) and 500 °C (red line) treated NiO films, where (**a**) presents the comparison of C 1s lines of the films annealed at 300 and 500 °C, (**b**,**c**) show the deconvoluted C 1s spectra. The black lines are presented the raw data, the pink lines are the contribution of the adventitious carbon. The studied NiO films have three layers deposited on ITO substrates.

Figure 4 presents the XPS profile of C 1s of the sol–gel NiO films, deposited on ITO and treated at 300 and 500 °C. The C 1s spectra were deconvoluted into two peaks centered at approximately 284.67 eV and 287.57 eV (300 °C annealed sample), and at 284.54 and 286.86 eV (500 °C). The peaks are assigned to non-oxygenated rings (C–C/C = C, 284.6 eV) that are related to adventitious carbon adsorbed on the sample surface and to C-O or/and C-OH bonds, 286–287 eV) [29]. The high-temperature treatment results in a reduced second line (at 286.36 eV).

**Figure 5:**
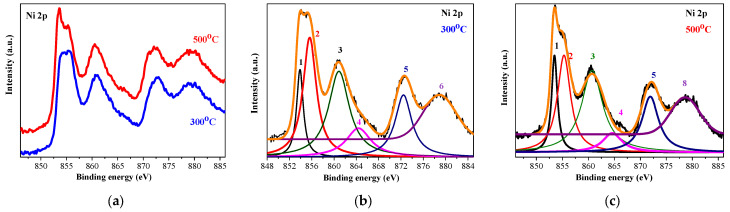
Ni 2p XPS spectra, where (**a**) shows the comparison of Ni 2p spectra of the NiO films annealed at 300 (blue line) and 500 °C (red line), and (**b**,**c**) show the deconvoluted Ni 2p spectra of 300- and 500 °C-treated films, respectively. The peak numbers and the corresponding color lines are described in the paper text. The studied NiO films have three layers deposited on ITO substrates.

Figure 5 depicts the high-resolution XPS spectra of Ni 2p of the two studied samples. Before proceeding further, each corrected XPS spectrum was subjected to a Shirley background subtraction procedure to remove the background caused by secondary electrons and photoelectrons that have lost energy during their escape from the sample. The comparison of the raw Ni 2p spectra is given in Figure 5a. The deconvoluted and the background-subtracted XPS Ni 2p spectra of the NiO films, treated at 300 and 500 °C, are demonstrated in Figure 5b and Figure 5c, respectively.

**Figure 6:**
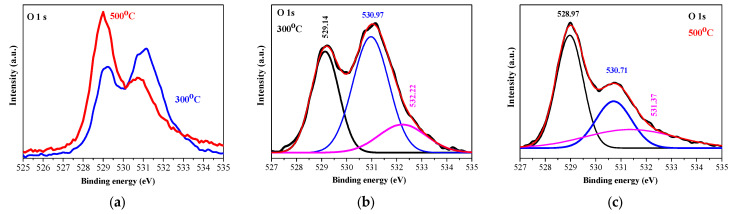
(**a**) The comparison of O1s XPS spectra of the NiO films annealed at 300 (blue line) and 500 °C (red line); (**b**,**c**) show the deconvoluted O 1s spectra of the 300 and 500 °C treated films, respectively. The black lines are the raw data and the pink colored lines are presented OIII lines, described in the text. The studied NiO films have three layers deposited on ITO substrates.

The film, treated at the lower temperature (Figure 5b), reveals the spin-orbit doublet, with peaks located at 853.5 eV (number 1) and 871.9 eV (number 5). These peaks are assigned to Ni 2p 3/2 and Ni 2p 1/2, respectively. They are separated by a spin-orbit splitting energy of 18.4 eV. This separation energy is indicative of Ni^2+^ in the NiO phase [29,30]. The difference value of 18.4 eV is larger than the theoretical value of 17.3 eV, and this can be attributed to the broad feature of the Ni 2p 1/2 peak and the observed line at 855.3 eV (number 2) in Figure 5 [31]. This doublet is accompanied by two satellite peaks at 860.7 eV (number 3) and 879.8 eV (number 6) [32]. Shake-up satellite peaks can originate from *d*–*d* transitions, which occur with the formation of a *2p* core hole and the associated charge transfer process, leading to core hole screening [33]. The peak at 855.3 eV (number 2 in Figure 5b) can be proposed as an indication for the presence of the Ni^3+^ state [31,34]. Proof of the existence of the Ni_2_O_3_ phase in the sol–gel films can also be seen from the O 1s spectra, shown in Figure 6. The O 1s peak at 529.14 eV is due to the Ni^2+^-O bond, and the peak at 530.97 eV is related to Ni^3+^ oxygen bondings [30].

The XPS analysis of the three-layered NiO films, obtained on ITO substrates and annealed at 300 °C, reveals Ni^3+^. This means that NiO films are not stoichiometric [30]. Ni^3+^ ions are induced by quasilocalized holes around Ni^2+^ vacancies in the lattice, and they generate p-type conductivity in the NiO*x* thin film [35].

Figure 5c exhibits the deconvoluted spectrum of Ni 2p of the 500 °C annealed film. The same peaks are observed. It is suggested that after the high-temperature annealing, the Ni^3+^ oxidation state is still present.

O 1s XPS core-level spectra (Figure 6a) show the difference between the two annealing temperatures. At the lower temperature, the O 1s spectrum is deconvoluted into three peaks (Figure 6b): 529.13 (OI), 530.97 (OII), and 532.32 eV (OIII), and the strongest intensity is detected for the OII peak. After 500 °C annealing, the three peaks are changed. The OI peak becomes predominant, the OII line is still intense, and the OIII peak appears as a shoulder (see Figure 6c). The peak at 529.5 eV is assigned to the lattice oxygen O^2−^ in NiO, corresponding to the Ni-O bond. The peak at 530.7 eV is related to the oxygen-containing species, and it can be associated with the presence of Ni_2_O_3_. The peak at 531–532 eV is attributed to defect states that are associated with Ni vacancies and/or to surface contamination [16]. XPS characterization reveals the existence of Ni^2+^ and Ni^3+^ bonding states in the sol–gel NiO films deposited on ITO substrates.

### 2.3. FTIR Study

**Figure 7:**
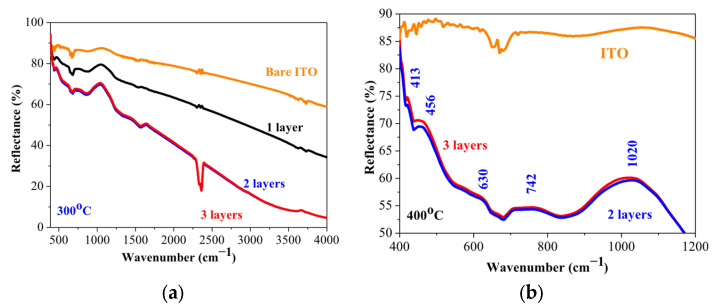
FTIR reflectance spectra of NiO films annealed at 400 °C, deposited on ITO substrates. (**a**) presents the spectra in the spectral range of 400–4000 cm^−1^ and (**b**) shows the enlarged view in the spectral range of 350–1200 cm^−1^. The spectrum of the bare substrate is given for comparison.

Figure 7 presents FTIR reflectance spectra of the sol–gel NiO films, obtained on ITO substrates. The samples are annealed at 400 °C. The spectrum of the bare ITO substrate is presented for comparison purposes. The observed bands, their positions, and their assignments are summarized in Table 2. The obtained results indicate that the sol–gel films are primarily NiO with the presence of defects and other nickel oxide phases. This is in agreement with XPS analysis, where the existence of the Ni^3+^ state is confirmed.

**Figure 8:**
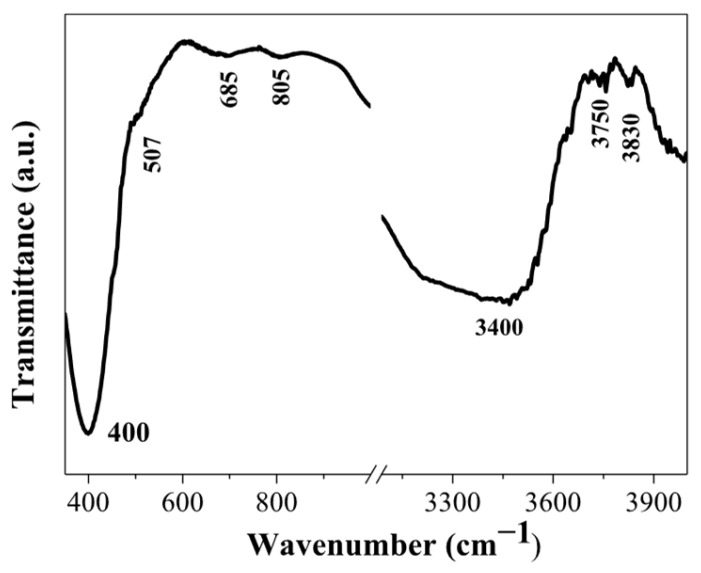
FTIR spectrum of the three-layered NiO film, annealed at 500 °C. The film is deposited on a silicon wafer.

FTIR spectrum of the three-layered NiO film, deposited on a Si wafer, is shown in Figure 8. The main absorption band is located at 400 cm^−1^, and a shoulder at 507 cm^−1^ is seen. These absorption bands are due to Ni-O bonding [36]. The broad absorption band in the spectral region of 600–700 cm^−1^ is assigned to the Ni-O stretching vibration mode; the broadness of the absorption band indicates that the NiO film has a nanocrystalline structure, as a similar feature is found in NiO nanocrystals [38]. The appearance of IR lines in the range of 445–540 cm^−1^ is assigned to the Ni-O stretching vibration mode and is clear evidence for the presence of the nanocrystalline NiO [37]. In this spectral range, IR bands due to Ni_2_O_3_ can also have a contribution to the absorption band, and the presence of this fraction cannot be excluded. The broad band at 805 cm^−1^ can be related to carbon impurities. Interesting features are seen in the spectral range 3000–4000 cm^−1^, where the hydroxyl group vibrations are revealed.

The broad band at 3400 cm^−1^ is due to the stretching vibration (υO-H) of the hydrogen-bonded hydroxyl group [39]. The absorption line at 3750 cm^−1^ is related to the stretching vibrations of the OH groups, which are often associated with adsorbed water molecules or with surface hydroxyl groups [39]. It must be pointed out that the weak IR lines above 3000 cm^−1^ (FTIR reflectance spectra of NiO films, obtained on ITO substrates, given in Figure 7a) can also be related to the surface-adsorbed hydroxyl groups.

### 2.4. FESEM Observation

**Figure 9:**
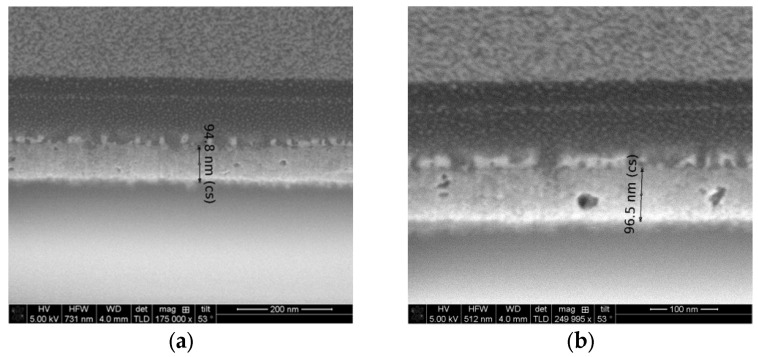
FESEM cross-section micrographs of the two-layered NiO films, deposited on glass substrates, where (**a**) is 300 °C annealed film and (**b**) is 400 °C annealed film.

FESEM micrographs of the two-layered NiO films, deposited on glass substrates, are presented in Figure 9. The image of the 300 °C annealed sample (Figure 9a) shows that the film thickness is 94.8 nm. The increase of the annealing temperature up to 400 °C very slightly changes the film thickness, and the value is 96.5 nm (see Figure 9b). From these micrographs, the presence of pores can be detected. It can be deduced that the sol–gel NiO films, deposited on glass substrates, possess a rather rough surface.

**Figure 10:**
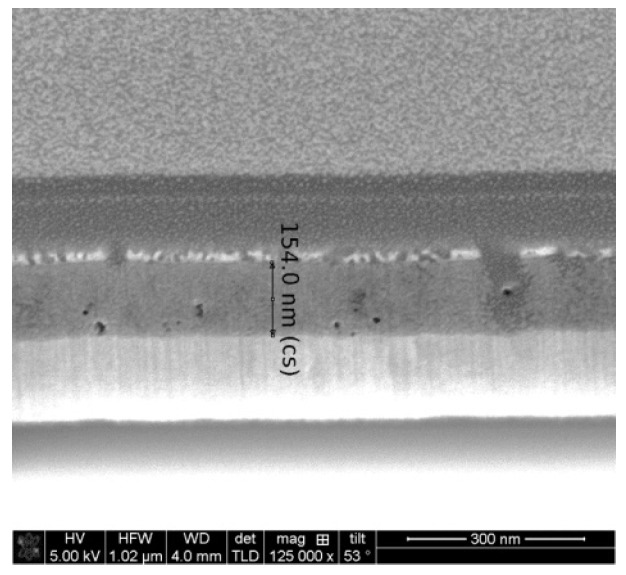
FESEM cross-section micrograph of the two-layered NiO film, obtained on an ITO substrate and treated at 300 °C.

The FESEM micrograph of the two-layered NiO film, deposited on an ITO substrate (see Figure 10), also reveals the appearance of pores and defects in the film structure. Surprisingly, the film thickness, assumed from the cross-section FESEM image, is up to 154 nm.

The FESEM study suggests that NiO films are deposited with different thicknesses on the different types of substrates, although the sol–gel spin-coating deposition was performed at the same time and at the same technological conditions.

### 2.5. Optical Properties

**Figure 11:**
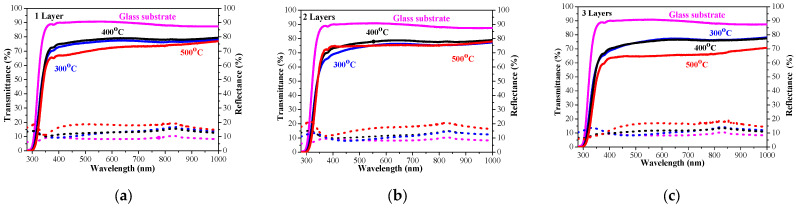
Transmittance (solid lines) and reflectance (dotted lines) spectra of NiO films, deposited on a glass substrate with (**a**) one layer, (**b**) two layers, and (**c**) three layers. The spectra are measured after the thermal treatments at 300 (blue color), 400 (black color), and 500 °C (red color). Transmittance and reflectance spectra of bare glass substrate are given for comparison (in magenta color).

**Figure 12:**
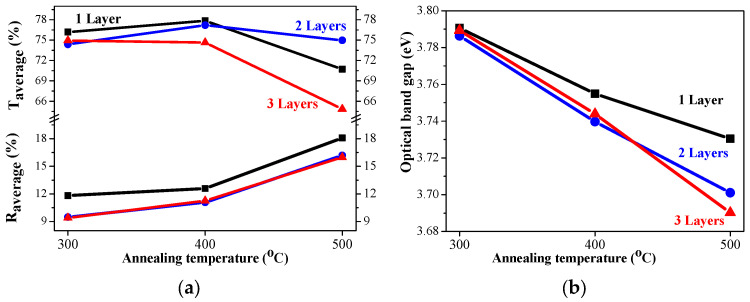
(**a**) The average transmittance and reflectance values of NiO films with different numbers of layers, where the data of the one-layered film are in black, the two-layered film is in blue, and the three-layered films are in red, as a function of the annealing temperature. The average value is calculated for the spectral range 450–700 nm; (**b**) The estimated optical band gap of NiO films, deposited on a glass substrate.

The sol–gel NiO films on glass substrates are optically characterized, and their transmittance and reflectance spectra are given in Figure 11. The transmittance spectra show good transparency in the range of 65–78%, strongly dependent on the number of layers and on the annealing temperatures. The NiO thin films with one and three layers have very similar transmittance spectra after annealing at temperatures of 300 and 400 °C. The high-temperature annealing at 500 °C results in a decrease in optical transmittance (see Figure 11a–c). The two-layered NiO films reveal a slight change in the optical transparency in the visible spectral range with the thermal treatments from 300 to 500 °C. The average transmittance values diminish with the increase in the number of layers in the spectral range of 400–700 nm (as seen from Figure 12a). The reduced transparency can be due to the light scattering of the rougher surface and the bigger crystallites. It is also reported that the presence of the Ni^3+^ oxidation state can decrease film transparency [34]. Ni^3+^ ions can be considered as color centers in the material [40]. The existence of Ni^3+^ states suggests the formation of interstitial oxygen and nickel vacancies in NiO films. The additional oxygen atoms in the film absorb photons, which reduces the film’s transparency [41]. Another reason for the transparency decrease can be due to the separation of NiO crystallites at the higher annealing temperatures and the formation of pores (empty spaces between them). This effect was observed by other researchers [42].

The optical band gap of the sol–gel NiO films was estimated by the first derivative of the transmittance dT/dλ [43]. The determined values are exhibited in Figure 12b. The band gap values of the NiO films deposited on glass substrates decreased with the increase in the number of layers and after annealing at higher temperatures. Optical band gap values are affected by several factors, such as grain size, film crystallinity, film composition, the presence of impurity ions in the host material, etc. [10]. The decreasing trend in the band gap is due to the improvement in film crystallinity and the growth of bigger crystallites, as confirmed by XRD observation (see Table 1).

**Figure 13:**
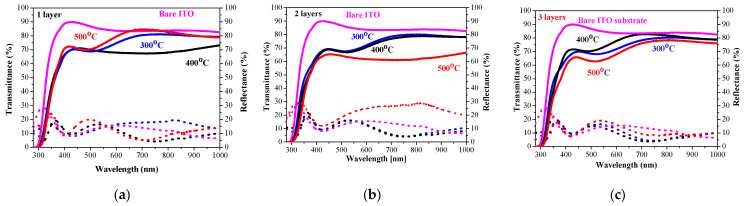
Transmittance (solid lines) and reflectance (dotted lines) spectra of NiO films, obtained on an ITO substrate with (**a**) one layer, (**b**) two layers, and (**c**) three layers. The spectra are measured after annealing at 300 (blue color), 400 (black color), and 500 °C (red color). Transmittance and reflectance spectra of the bare ITO substrate are given for comparison (in magenta color).

The measured transmittance and reflectance spectra of the one-, two-, and three-layered NiO films, deposited on ITO substrates, are presented in Figure 13. With the exception of one-layered film (Figure 13a), NiO films with two and three layers (Figure 13b,c) manifest a decrease in the film transparency after annealing at 500 °C.

**Figure 14:**
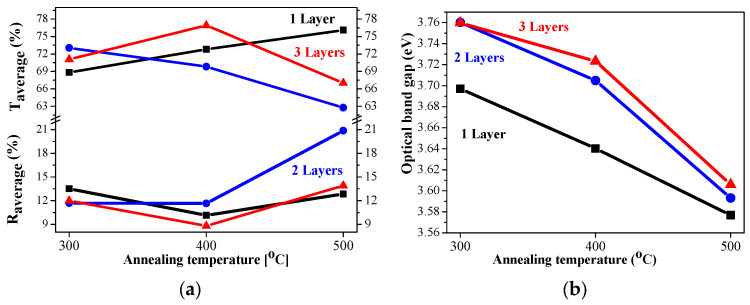
(**a**) The average transmittance and reflectance values of NiO films, obtained on ITO substrates with different numbers of layers, where the data of the one-layered film are in black, the two-layered film is in blue, and the three-layered films are in red, as a function of the annealing temperature. The average value is calculated for the spectral range 450–700 nm; (**b**) the estimated optical bandgap of NiO films, deposited on an ITO substrate.

The average transmittance values are in the range 63–78% and the reflectance values are below 20% (Figure 14a). A comparison of the transparency of the three-layered films deposited on ITO substrates with the films obtained on glass substrates leads to interesting findings. NiO films deposited on ITO-covered glass become more transparent after 400 and 500 °C annealing in the spectral range above 600 nm than the NiO films deposited on glass substrates. Their average values in the visible spectral range are 74.6% (400 °C) and 64.9% (500 °C) of NiO films on glass substrates, and corresponding values in the case of the ITO substrate are 76.9 and 66.7% after 400 °C and 500 °C annealing. The corresponding average transmittance values of the bare substrates are 90.31% (glass) and 85.72% for the ITO substrate. The film thickness of the samples, deposited on ITO substrates, is higher, and the crystallites are bigger, as concluded from FESEM and XRD studies. The greater transparency can be due to the smoother surface morphology and the decreased pore density of NiO films, obtained on ITO substrates. It is interesting to note that the three-layered NiO films on ITO substrates possess the best transparency among the studied films after annealing at 400 °C.

The optical bandgap of NiO films, deposited on ITO substrates, varies from 3.76 eV to 3.58 eV, and the values are almost linearly decreasing with increasing annealing temperature, as shown in Figure 14b. The optical bandgap values are strongly correlated with the number of layers. The determined values of the sol–gel NiO films are in the range of the reported optical bandgap data of the NiO films [44,45,46].

### 2.6. Electrical Properties of Sol–Gel NiO Films

#### 2.6.1. Sheet Resistance

The sheet resistance values are measured by using the four-point probe method of NiO films obtained on the glass substrates. The sheet resistance measurements are with an accuracy of 1%. The sol–gel NiO films, deposited on glass, are conductive with the sheet resistance values from 691 to 168 Ω/□. Based on the sheet resistance measurements and XPS results, it is proposed that the films possess p-type conductivity. The sheet resistance strongly depends on the number of layers and the thermal treatments. The obtained data are given in Table 3. The results are within and even better than the reported values [47,48]. In reference [47], the reported sheet resistance values varied from 287.8 to 743.2 Ω/□ for NiO films with the film thickness changed from 171 to 223 nm.

One-layered NiO films reveal a sharp decrease in the sheet resistance after 500 °C annealing. The sol–gel two-layered samples have also manifested that sheet resistance is decreased with increasing annealing temperatures. This indicates that the film conductivity is enhanced. The thickest films show the lowest sheet resistance at the low temperature treatment at 300 °C, and the values are increased after higher temperature annealings. The improvement in the conductivity of NiO films can be provoked by the existence of the interstitial oxygen and nickel vacancies [49], as well as the presence of the Ni^3+^ oxidation state [35]. In the case of the three-layered NiO films, the annealing at the highest temperature leads to a lowering of the oxygen peak, which is assigned to Ni^3+^ (XPS spectra, Figure 6), and the conductivity is reduced.

The figure of merit (*FOM*) gives the relation between the optical transparency and the sheet resistance of conductive thin films in order to find a way to compare the properties and the quality of transparent conducting films, obtained by various methods with different film thicknesses. FOM values are determined by the Haacke relation by using the measured sheet resistance (*R_sheet_*) and the average transmittance (*T_average_*) values in the visible spectral range. The relation is introduced by Haacke [50]:

(1) $$FOM=\frac{T_{average}^{10}}{R_{sheet}}$$

The estimated *FOM*s are presented in Table 3. The maximum *FOM* values are obtained for the three-layered NiO film, annealed at 300 °C. The reported *FOM* values vary in the range of 4 × 10^−13^, 1.1 × 10^−10^, and up to 1.35 × 10^−7^ Ω^−1^ for doped NiO films [47,51,52]. The obtained sol–gel derived NiO films are very encouraging.

#### 2.6.2. Work Function (WF) of Sol–Gel NiO Films, Deposited on Si, Glass, and ITO Substrates

**Figure 15:**
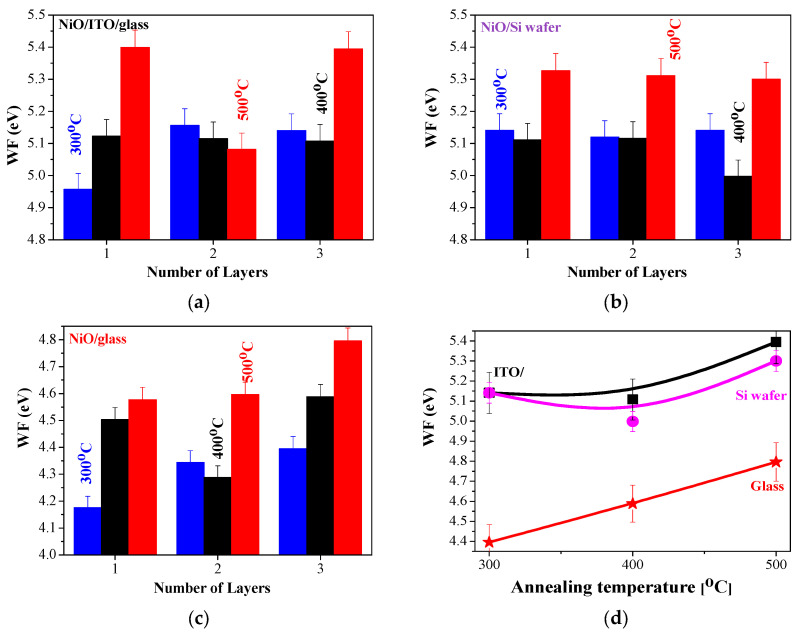
WF values of sol–gel NiO films, deposited on (**a**) ITO substrate, (**b**) Si wafer, and (**c**) glass substrate; (**d**) presents the comparison of the three-layered NiO films, deposited on glass (red color), silicon (magenta), and ITO (black color) substrates as a function of the annealing temperatures.

WF values of NiO films are reported in a wide range from 3.8 to 6.7 eV [53,54,55]. The work function (WF) of NiO_x_ thin films can be varied due to factors like deposition methods, technological conditions, defects (oxygen vacancies or surface impurities), composition, and crystal structure [54,56]. It is also reported that the work function of NiO can be enhanced by increasing the nickel vacancies and excess oxygen [57]. Figure 15 presents the work function values measured for the sol–gel NiO films deposited on different substrates. The influence of the number of layers and the annealing temperatures is also given.

The sol–gel NiO films show the highest WF values for 500 °C annealed samples. Similar findings are reported for chemical bath-deposited NiOx films, with the reported WF values being 4.7, 5.25, and 5.48 eV after annealing at 200, 300, and 400 °C, respectively [54]. The enhanced work function of NiO films, obtained by different deposition methods with thermal treatments, is also announced [17]. The obtained data are within the frames of the reported values [17,54,55,56,57,58].

It is also interesting to study the substrate effect on the WF values of sol–gel NiO films. The substrate influences the growth of NiO films, thus it reflects on their properties. It has been stated [58] that the evaporated NiOx film on the Si substrate reveals a variation in WF values due to air exposure and the formation of NiSi_x_ near the interface with the Si wafer. The sol–gel samples on Si wafers show slightly lower WF values compared to NiO films obtained on ITO substrates. As is seen (Figure 15c), the lowest WF values are obtained for the sol–gel NiO films deposited on glass substrates. XRD study manifests that the preferential orientation of NiO films depends strongly on substrate type—glass or ITO. The lower WF values of the NiO/glass samples can be explained by the smaller crystallite sizes (see Table 1), as the smaller grain size can decrease the work function due to the higher concentration of grain boundaries [53]. The preferential crystal orientation of these samples is in the (200) plane. It is reported that the (111) NiO possesses an increased work function [55] (such preferential growth of the crystallites is found for NiO/ITO samples). The crystalline structure and the preferential growth of the crystallites affect the obtained WF values.

**Figure 16:**
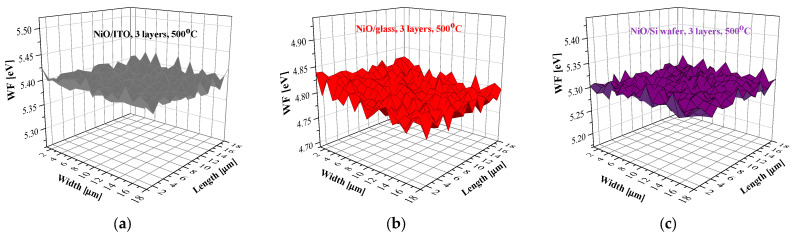
WF mapping of the three-layered NiO films, deposited on (**a**) ITO, (**b**) glass, and (**c**) Si substrates. The measured samples were annealed at 500 °C.

Figure 16 presents the mapping of the WF of the three-layered NiO films, deposited on the three types of substrates after annealing at 500 °C. The WF value of the ITO substrate is 5.2 eV, and the Si wafer has a WF = 4.35 eV. The sol–gel films reveal closed WF values within the measured area.

The obtained results reveal that the work function of sol–gel NiO films is sensitive to the substrate type, film thickness, and thermal treatments.

The knowledge of the work function of NiO films is essential for their performance in electronic and optoelectronic devices, including solar cells, sensors, etc. In photovoltaics, the work function measurement is important to assess electronic band offsets (i.e., band alignment) between the different layers in the solar cell, which determine the efficiency of charge transport in the device [59]. For example, the difference in work function between the p-type and n-type semiconductors in a p-n heterojunction creates an electric field at the interface that separates photogenerated electron–hole pairs, improving efficiency in applications like photocatalysis [60]. The application of NiO films as hole transport layers depends on the wide bandgap around 3.6 eV (transparent in the visible spectral region) and a tunable work function between 4.8 and 5.4 eV [18]. WF influences the band alignment between NiOx and the active layer of the solar cell, ensuring that holes are easily extracted from the active layer and that electrons are blocked [18]. The obtained work function values of the sol–gel NiO films show promise for applying these films in solar cell applications.

## 3. Conclusions

Thin films of conductive NiO films were successfully obtained by the sol–gel spin-coating method on Si, glass, and ITO substrates. The XRD study proves the formation of cubic NiO as the nickel oxide film is preferentially grown in a (200) orientation when it is coated on a glass substrate. The sol–gel NiO films deposited on an ITO substrate are crystallized in the predominant (111) direction. The XPS investigation reveals that the sol–gel NiO films are not stoichiometric, and the Ni^3+^ state presence is found. The XPS results and the four-point probe measurements propose that sol–gel NiO films can be p-type semiconductors. The FTIR spectroscopy confirms the XRD conclusions. The films deposited on glass substrate are transparent by up to 77.5%, and the NiO films/ITO substrates show transmittance up to 76.3% in the visible spectral range. The thermal treatments and the film thickness slightly affect the transparency of NiO films in the spectral range of 450–700 nm. The optical bandgap is very sensitive to the thermal treatments and the number of layers. Work function values of NiO films, deposited on Si and ITO substrates, are in the range of 5.0 to 5.4 eV. The three-layered NiO films on ITO substrates possess very good transparency (they are better than the two-layered samples and close to that of one-layered films) with good electrical properties (high work function values). These properties can lead to the suggestion that NiO films, deposited at these technological conditions, are the most promising materials. The three-layered NiO films on glass substrates show low sheet resistance and good FOM values. The results of studying the optical, structural, and electrical properties revealed that sol–gel NiO thin films exhibit satisfactory characteristics for photoelectric device applications.

## 4. Materials and Methods

The sol solution synthesis had been previously described [19].

The source material for the nickel-containing solution was nickel acetate (Ni(CH_3_COO)_2_ × 4H_2_O, Alfa Aesar, Ward Hill, MA, USA, 98+% pure). The used solvent was an absolute ethanol (Merck, Darmstadt, Germany, absolute for analysis). The prepared sol solution had a 0.4 M concentration.

The solution was stirred at a temperature of 60 °C for thirty minutes by using a magnetic stirrer (ARE model, Velp Scientifica Srl, Usmate, Italy). Monoethanolamine (MEA, Fluka AG, Buchs, Switzerland, 98% purity) was introduced in order to achieve a molar ratio MEA/Ni equal to 1. The monoethanolamine acted as a complexing agent and stabilizer. The solution was stirred for 15 min at 60 °C/15 min and then ultrasonically treated at 40 °C for 15 min (UST 2.8-100 ultrasonic cleaner, Siel Ltd., Gabrovo, Bulgaria). The sol solution was kept for a 24 h aging. The synthesized nickel solution was clear and homogeneous. There was no precipitate, and the solution possessed good film-forming capabilities.

The nickel oxide films were spin-coated (P 6708 spin coater, PI-KEM Limited, Staffordshire, UK) at a rotation speed of 2000 rpm/30 s. The preheating temperature was 300 °C for 10 min. The preheating temperature was the initial heating step applied between layers and before final film sintering, and it had a crucial role in the film formation. The layer deposition and preheating sequence were repeated until the desired number of layers was achieved. The studied NiO films were with 1, 2, and 3 layers. Afterwards, the films were annealed at various temperatures ranging from 300 to 500 °C for 60 min in ambient air. The two heat treatment processes were in a chamber furnace (Tokmet TK Ltd., Varna, Bulgaria). The furnace’s constant heating and cooling rates were 10 °C/minute. Three types of substrates were used: high-purity silicon wafers produced by the float zone (FZ) method, doped to be a p-type semiconductor, with a resistivity 4.5–7.5 Ω and a crystal orientation of <100; soda-lime glass substrates; and 25 × 25 mm ITO (indium tin oxide)-coated glass slides with a resistance of <15 Ω and a standard thickness of 1.1 mm, produced by Ossila Ltd., Sheffield, UK).

The film crystallization was studied by using a Bruker D8 Advanced diffractometer (Bruker Cooperation, Karlsruhe, Germany) with a copper source (CuKα), and the diffraction patterns were measured in an angular range (2θ from 35° to 90°) with a constant scan rate of 0.02°/s. XPS analyses were carried out applying a Kratos AXIS Supra spectrometer (Kratos Analytical, Manchester, UK) with an achromatic Al X-ray source and ultra-high vacuum (<10^−8^) at a 90-degree take-off angle. Each analysis started with a survey scan performed from 0 to 1200 eV. The pass energy was 160 eV at steps of 0.5 eV with one sweep. The number of sweeps was increased in order to perform high-resolution analysis as well, and the pass energy was decreased to 20 eV at steps of 100 meV. The carbon 1s core-level peak at 284.6 eV was used for the energy calibration of photoelectron measurements of the samples studied. FTIR measurements were made using the IRPrestige-21 spectrophotometer (Shimadzu Corporation, Kyoto, Japan). The transmittance spectra were taken in the spectral range from 350 to 4000 cm^−1^. A bare S) wafer was used as a background. The specular reflectance attachment was used to measure the reflectance spectra of the sol–gel films deposited on ITO substrates. The aluminum mirror was applied to establish the baseline. Optical characterization was realized by using a Shimadzu UV–VIS–NIR 3600 spectrophotometer (Shimadzu Corporation, Kyoto, Japan), and the measurements were done in the spectral region spanning from 280 to 1800 nm. The background for the transmittance spectrum was taken using air. The reflectance spectra were measured by using the 5° specular reflectance accessory and aluminium-coated mirror as a reference standard. Work function (WF) of the samples was measured with a Scanning Kelvin Probe (SKP5050, KP Technology Ltd., Wick, Caithness, UK). The Kelvin probe measures the contact potential difference between a sample and a tip. The WF of the sample was calculated by determining the WF of the tip calibrated against a known surface. Cross-section samples were prepared by focused ion-beam etching using FEI’s Dual Beam Helios Nanolab system. SEM was done using a Zeiss Supra 40 (Carl Zeiss AG, Oberkochen, Germany).

## Figures and Tables

**Table 1:** XRD data and the calculated crystalline sizes of sol–gel NiO films deposited on glass and ITO substrates.

Substrate	Number of Layers	Annealing (°C)	2θ Degrees	Miller Indices	FWHM	Crystallite Size (nm)
Glass	3	400	37.21 43.13	111 200	Weak, broad 1.15049	- 7.58
	3	500	37.42 43.35	111 200	2.07068 0.6358	4.23 13.73
ITO	3	300	37.36	111 200	0.69502 -	12.60 -
	3	400	37.44 43.35	111 200	0.33477 broad	26.18 -
	3	500	37.42 43.36	111 200	0.42876 0.92663	20.42 9.52
	2	400	37.21 43.13	111 200	0.28302 broad	30.33 -

**Table 2:** FTIR data of the sol–gel NiO films annealed at 400 °C, deposited on ITO substrates.

IR Line (cm^−1^)	Assigned to
413	Ni-O stretching mode [36]
459	stretching vibration Ni-O, NiO, or Ni_2_O_3_ [37]
630	defects and surface oxygen interstitials from Ni-O vibrations [38]
742	Ni-O stretching vibration [38]
1020	C-O modes due to the deposition process [19]

**Table 3:** Sheet resistance (*R_shee_*_t_), average transmittance (T_average_ in the spectral range of 450–700 nm), and the estimated figure of merit (*FOM*) of sol–gel NiO films, with different numbers of layers and after annealing at 300–500 °C. The substrate used is glass.

Number of Layers	Annealing Temperature (°C)	*R_sheet_*_,_ (Ω/□)	*T_average_* (%) λ = 450–700 nm	Figure of Merit ×10^−5^ (Ω^−1^)
1	300	688	76.18	9.56
	400	691	77.85	11.83
	500	220	70.71	14.20
2	300	377	74.36	13.71
	400	255	77.19	29.45
	500	242	74.97	23.18
3	300	168	74.93	33.21
	400	235	74.64	22.84
	500	250	64.85	5.26

## Data Availability

The original contributions presented in this study are included in the article. Further inquiries can be directed to the corresponding author.
